# An Altered Microbiota in the Lower and Upper Female Reproductive Tract of Women with Recurrent Spontaneous Abortion

**DOI:** 10.1128/spectrum.00462-22

**Published:** 2022-05-23

**Authors:** Fen-Ting Liu, Shuo Yang, Zi Yang, Ping Zhou, Tianliu Peng, Jingwen Yin, Zhenhong Ye, Hongying Shan, Yang Yu, Rong Li

**Affiliations:** a Center for Reproductive Medicine, Department of Obstetrics and Gynecology, Peking University Third Hospital, Beijing, China; b National Clinical Research Center for Obstetrics and Gynecology, Peking University Third Hospital, Beijing, China; c Key Laboratory of Assisted Reproduction, Peking University, Ministry of Education, Beijing, China; d Beijing Key Laboratory of Reproductive Endocrinology and Assisted Reproductive Technology, Peking University Third Hospital, Beijing, China; University of Nebraska-Lincoln

**Keywords:** cervical microbiota, inflammation, recurrent spontaneous abortion, uterine microbiota, vaginal microbiota

## Abstract

Recurrent spontaneous abortion (RSA) is a complex multifactorial disease. Recently, the microbiota of the female reproductive tract, as an emerging factor in RSA, has gradually attracted the attention of many clinical researchers. Here, we reported that the microbiota of the lower and upper female reproductive tracts from patients with RSA showed no significant differences in alpha diversity compared to that of controls. Beta diversity was significantly higher in the RSA group than in the control group in the vaginal microbiota (*P* = 0.036), cervical microbiota (*P* = 0.010) and microbiota from uterine lavage fluid (*P* = 0.001). In addition, dramatic decreases in gamma interferon and interleukin-6 cytokine levels were observed in the RSA group. In conclusion, our data suggested altered microbial biodiversity in the vagina, cervix and uterine lavage fluid in the RSA group. Alterations in the microbiota in the uterine cavity could be associated with altered cytokine levels, which might be a risk factor for RSA pathogenesis. Moreover, the microbiota composition differed markedly from the lower genital tract to the uterine cavity, and the microbiota in the uterine cavity also distinctly varied between endometrial tissue and uterine lavage fluid in the RSA group. Hence, sampling with these two methods simultaneously allowed a more comprehensive perspective of microbial colonization in the uterine cavity.

**IMPORTANCE** As an obstacle to pregnancy, recurrent spontaneous abortion (RSA) can be caused by a variety of factors, and a current understanding of the etiology of RSA is still lacking; half of cases have an unknown cause. A substantial fraction of patients show no improvement after treatment. Since the microbiota of the female reproductive tract has been proposed as an emerging factor in RSA patients, further investigation is needed to provide guidance for clinical therapy. In general, this is the first report describing the distinct alterations of the vaginal, cervical, and uterine microbiota in RSA, not just that in the vagina. Furthermore, another major strength of this study derived from the further in-depth investigation and analysis of the characteristics of the microbiota colonizing the upper female genital tract in RSA, which provided a more comprehensive view for investigating the uterine microbiota.

## INTRODUCTION

The definition of recurrent spontaneous abortion (RSA) mostly refers to two or more consecutive miscarriages before 20 weeks, and 1–3% of childbearing women suffer from this situation (1). An obstacle to pregnancy, RSA can be caused by a variety of factors, including genetics, uterine anomalies, autoimmune diseases, and infection (2). However, the current understanding of the etiology of RSA is still lacking, and half of the cases have unknown causes (3). Although preimplantation genetic testing in assisted reproduction technology can reduce the abortion rate of RSA, a substantial fraction of patients show no improvement (4). Therefore, it is imperative to further investigate the potential etiologies and treatment strategies. With rapid advances in sequencing technologies, a growing number of microorganisms colonizing the female genital tract can be discovered and recognized (5). Evidence suggests that the compositions and proportions of the microbiota throughout the female genital tract have important implications for fertility (6, 7). A healthy microbiota in the vagina dominated by *Lactobacillus* can prevent infection, and a decrease in its abundance might be associated with infertility and adverse pregnancy outcomes in *in vitro* fertilization (IVF), with undefined mechanisms (6, 8). The endometrial microbiota has a complex and diverse composition, and its imbalance is closely related to infertility and the embryo implantation process (6, 9). The characteristics of the microbiota harbored in vaginal secretions in RSA have been revealed in several studies. Studies have demonstrated that two genera (Atopobium and Prevotella) exhibited significantly greater abundance, along with higher expression of CCL2/CCL8/CCL3/CCL4/CCL5, in RSA patients (10–13). However, the abundance of Gardnerella in RSA patients remains controversial (10, 13). A recent study indicated that eight additional taxa were detected in endometrial fluid but not in endometrial tissue. In total, 20-two taxa showed markedly different abundances between fluid and tissue samples in patients with recurrent miscarriage (14). However, most studies focus on the impact of the vaginal microbiota (VM) due to methodological limitations of sampling, and less attention has been given to the cervical and uterine microbiota in RSA patients. There remains a lack of systematic and complete research on the alterations in and role of the microbiota in the lower and upper female reproductive tracts of RSA patients. Moreover, imbalanced expression of Th1/Th2/Th17 cytokines and significant elevation of interferon (IFN)-γ, tumor necrosis factor (TNF)-α, interleukin (IL)-6, and IL-17A levels in Chlamydia trachomatis (Ct)-positive recurrent miscarriage patients have been observed (15). Hence, the alterations in the vagina, cervix, and uterine microbiota in RSA patients and whether these alterations in the uterine cavity lead to a subsequent change in cytokine levels remain to be fully elucidated. In this study, the composition and diversity of the vaginal, cervical, and uterine microbial communities in patients with RSA were systematically analyzed, and Th1/Th2/Th17 cytokine levels in the uterine cavity were also measured to further determine the potential etiology of RSA.

## RESULTS

### Clinical information of the included patients.

To obtain a comprehensive view of microbiota alterations in RSA patients, samples of 25 RSA and 25 control patients were included for analysis. The basic clinical information of the included patients in this study was analyzed and is shown in Table 1. There were no differences in age, BMI, menstrual cycle, or basal sex hormone levels between the groups, which indicated that these two sets of clinical data were basically matched in this study. Of note, there was a significant difference in the proportion of patients with chronic endometritis between these two groups. pH tests of vaginal secretions (Figure S4a in the supplemental material) and cervical canal secretions (Figure S4b in the supplemental material) via pH meter, and uterine lavage fluid via pH test strips (Figure S4c) showed no significant differences in the control and RSA groups.

**TABLE 1:** Clinical characteristics of enrolled patients whose samples were collected and further processed for microbiota sequencing^*a*^

Variables	RSA (*n *=* *25)	CON (*n *=* *25)	*P* value
Basic information			
Age (yrs)	32.56 ± 3.50	31.60 ± 3.49	0.336
BMI (kg/m^2^)	22.19 ± 3.49	21.46 ± 2.93	0.429
Menstrual cycle	28.34 ± 2.85	29.44 ± 2.51	0.154
Basal sex hormone level			
FSH (mIU/mL)	4.17 ± 3.50	4.10 ± 3.85	0.947
LH (mIU/mL)	4.00 ± 3.29	4.09 ± 2.97	0.922
P (nmol/L)	29.40 ± 13.72	29.80 ± 16.94	0.927
E_2_ (pmol/L）	484.75 ± 346.62	369.40 ± 194.10	0.155
AMH (ng/mL）	3.34 ± 2.17	3.07 ± 1.65	0.638
Chronic endometritis	13 (52.0%)	6 (24.0%)	0.041*

a BMI: body mass index; FSH: follicle-stimulating hormone; LH: luteinizing hormone; P: progesterone; E2: estrogen; AMH: anti-mullerian hormone. * *P* <0.05.

### Comparison of the diversity and composition of the microbiota between the RSA and control groups.

**FIG 1:**
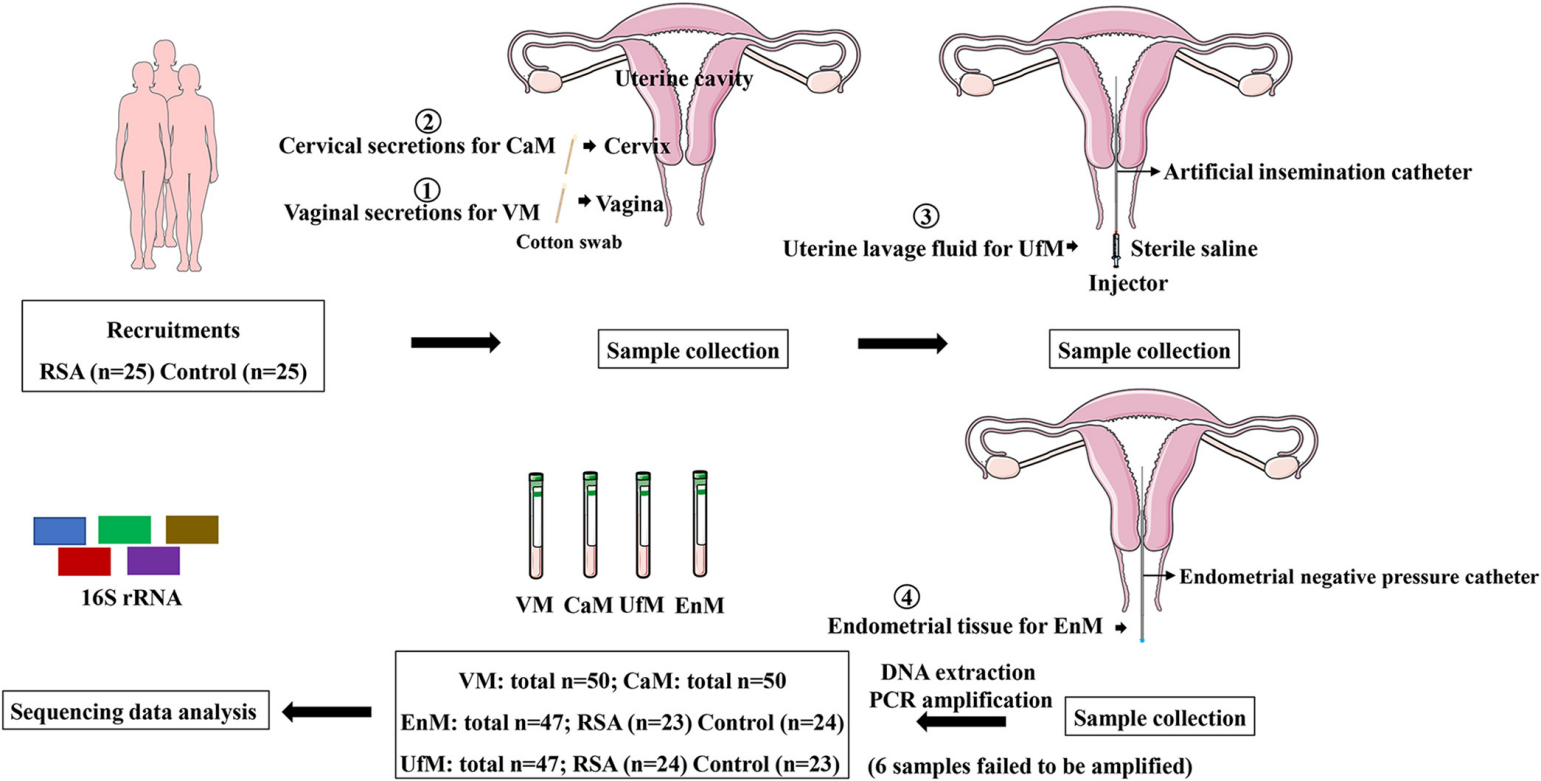
Schematic illustration of the study design.

**FIG 2:**
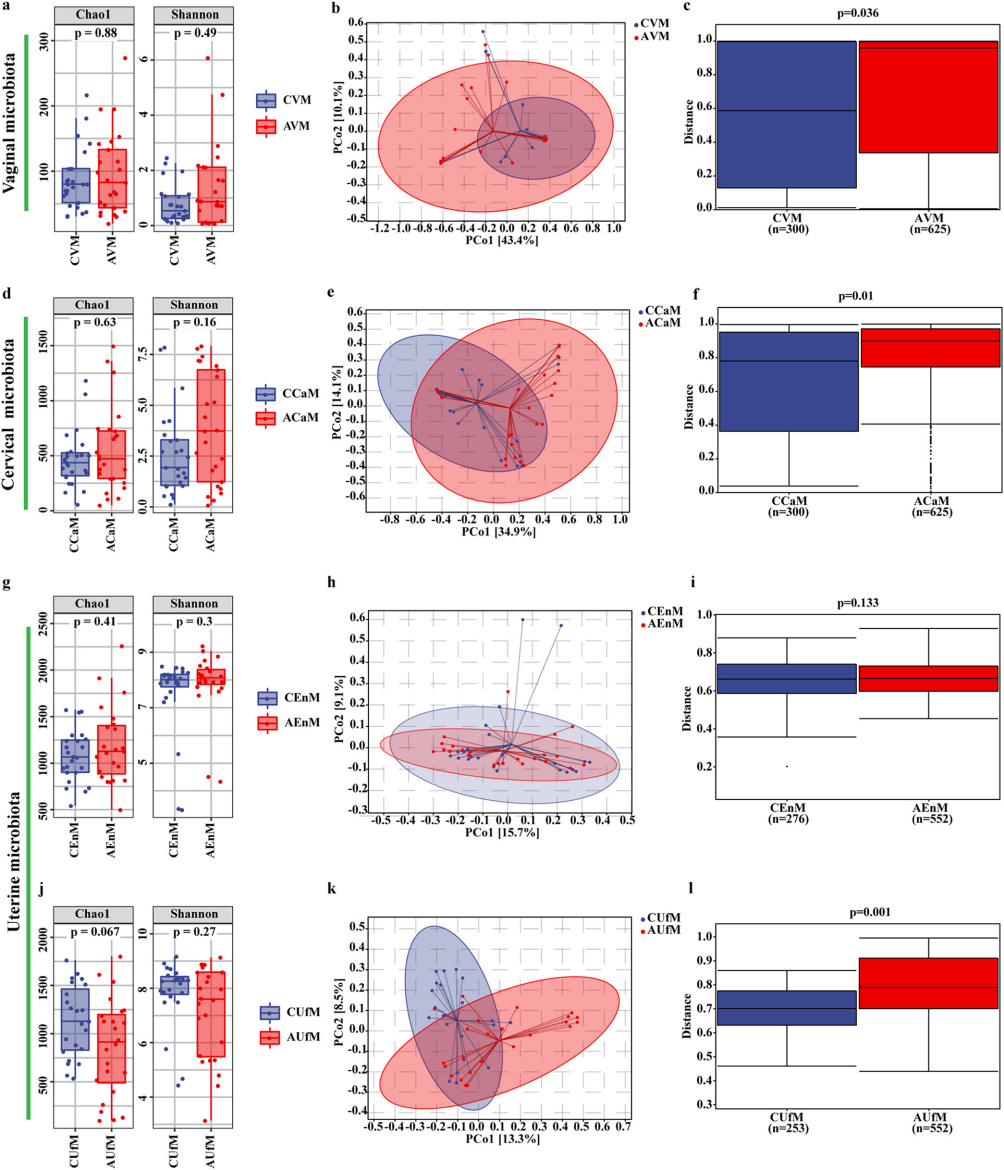
Comparison of the alpha and beta diversity of the microbiota in the vagina, cervix and uterine cavity between the RSA and control groups. Alpha diversity including Chao 1 and Shannon index (a, d, g, j) of different samples in the two groups. PCoA plots (b, e, h, k) from the control and RSA groups. Each point in PCoA plots represents one sample. Beta diversity based on Bray–Curtis metrics of the control and RSA groups (e, f, i, l). The upper and lower lines of the box represent the upper and lower interquartile range (IQR); horizontal lines represent the medians; upper and lower edges, maximum and minimum (extremum within 1.5 times IQR range); points on the outside of the upper and lower edges represent outliers. *P* values were determined by a two-tailed Mann–Whitney U test. Data are presented as medians with IQRs.

Six uterine cavity samples were excluded due to DNA extraction issues. In total, 194 samples were analyzed by 16S rRNA gene sequencing (Fig. 1). A total of 13,888,867 usable reads were obtained and analyzed, and a total of 21,545 OTUs were delineated at a 97% similarity level. Alpha diversity analysis was applied to assess the richness, diversity, and evenness of the microbiota within the different environments. All comparisons of different samples in the RSA and control groups did not detect any significant difference in the Chao1 or Shannon index (Fig. 2a, d, g, and j). The rarefaction curves of the sequenced samples tended to a constant level, which suggested that sufficient sequencing depth was obtained (Fig. S1 in the supplemental material).

### Comparison of the vaginal microbiota (VM) between the RSA and control groups.

**FIG 3:**
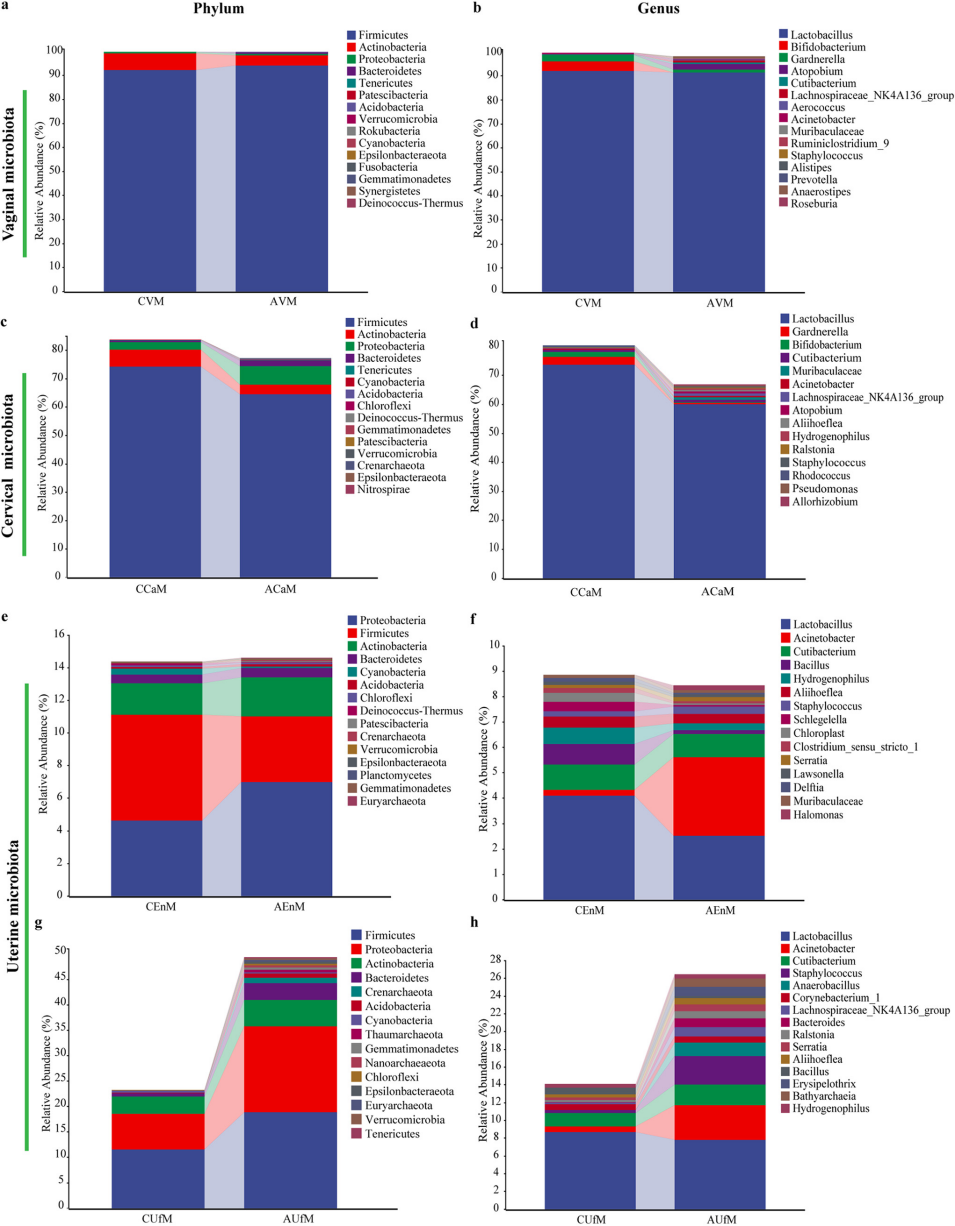
Taxonomic classification of the vaginal, cervical and uterine microbiota at the phylum and genus levels from the control and RSA groups.

Principal coordinate analysis (PCoA) was used to determine the trends of variation in the microbiota between the two groups via dimensionality reduction, and each dot distributed within the figure represents a single sample. Slight separation of these two groups in the VM was observed, and PCo1 and PCo2 accounted for 43.4% and 10.1% of the variation, respectively (Fig. 2b). To determine the significance of the distribution of differences between groups of samples, permutational multivariate analysis of variance (PERMANOVA) based on Bray–Curtis dissimilarity was used. A significantly higher beta diversity of the VM in the case group than in the control group (*P* = 0.036) was demonstrated (Fig. 2c). The taxonomic composition of the microbiota was investigated at the phylum and genus levels in the different sample communities, and the average relative abundances of the corresponding bacterial taxa were compared. For the VM at the phylum level, Firmicutes (92.15% ± 22.49% versus 94.03% ± 18.99%, *P* = 0.367) was the dominant phylum in both groups. Actinobacteria (6.99% ± 22.22% versus 4.29% ± 17.18%, *P* = 0.528), the second dominant phylum in the vagina, was slightly decreased in abundance in the RSA group (Fig. 3a and Table S1). Lactobacillus (92.06% ± 22.49% versus 91.28% ± 27.12%, *P* = 0.421) was the dominant genus in the vagina in both groups. Compared with those in the RSA group at the genus level, two bacterial taxa, Bifidobacterium and Gardnerella spp., showed a higher relative abundance in the control group, but the difference was not significant (Fig. 3b and Table S2 in the supplemental material).

### Comparison of the cervical microbiota (CaM) between the RSA and control groups.

Moderate separation of these two groups in the CaM was observed. PCo1 and PCo2 accounted for 34.9% and 14.1% of the variation, respectively (Fig. 2e). The result in Fig. 2f also demonstrated a significantly higher beta diversity of the CaM in the case group than in the control group (*P* = 0.010). Similar to the VM, Firmicutes and Actinobacteria were also the top two most important phyla in the CaM in both groups. Both Firmicutes (74.18% ± 30.35% versus 64.42% ± 32.31%, *P* = 0.265) and Actinobacteria (5.96% ± 15.67% versus 3.37% ± 6.85%, *P* = 0.528) showed higher relative abundances in the control group, while Proteobacteria (2.67% ± 3.91% versus 6.60% ± 7.31%, *P* = 0.051) showed a higher relative abundance in the RSA group. Bacteroidetes (0.54% ± 1.22% versus 2.00% ± 4.81%, *P* = 0.011) and Crenarchaeota (0% ± 0.01% versus 0.04% ± 0.09%, *P* = 0.010) showed distinctly higher abundances in the RSA group (Fig. 3c and Table S1 in the supplemental material). Similar to the VM at the genus level, Lactobacillus, Bifidobacterium, and Gardnerella spp. also showed higher relative abundances in the control group, but the differences were not significant, while the rest of the other bacterial taxa, including Cutibacterium (0.59% ± 0.78% versus 0.95% ± 1.01%, P = 0.045), Atopobium (0.04% ± 0.22% versus 0.76% ± 3.77%, *P* = 0.007), and Staphylococcus spp. (0.14% ± 0.35% versus 0.30% ± 0.60%, *P* = 0.043), showed a significant tendency toward increasing abundance in the RSA group (Fig. 3d and Table S2).

### Comparison of the uterine microbiota between the RSA and control groups.

While the microbiota of endometrial tissue (EnM) highly overlapped, significant separation of the PCoA results for the microbiota of uterine lavage fluid (UfM) was found between the RSA and control groups (Fig. 2h and k). The beta diversity results further suggested that only the UfM showed remarkable differences between the case group and the control group (*P* = 0.001), but no statistically significant changes in the EnM were observed (*P* = 0.133) (Fig. 2i and l). For the EnM, Proteobacteria (4.61% ± 3.86% versus 6.97% ± 14.52%, *P* = 0.782) became the predominant phylum in the RSA patients, while Firmicutes (6.51% ± 10.48 versus 4.05% ± 4.46%, *P* = 0.610) still played a dominant role in the control group (Fig. 3e and g, Table S1). Lactobacillus spp. still represented the major constituent but with a remarkable decrease in abundance in the uterine cavity, and the uterine cavity gradually turned into a multimicrobial environment. Proteobacteria was a large phylum including Acinetobacter, Hydrogenophilus, Schlegelella, Serratia, Delftia, and so on. Instead of Lactobacillus, Acinetobacter spp. (3.11% ± 13.68% versus 0.24% ± 0.27%, *P* = 0.663) belonging to the Proteobacteria became the predominant genus in RSA group (Fig. 3f and Table S2 in the supplemental material). Lactobacillus spp. (4.08% ± 10.06% versus 2.51% ± 4.00%, *P* = 0.882) in the EnM showed a tendency toward lower abundance in the RSA group (Fig. 3f and Table S2). For the UfM, Firmicutes (11.55% ± 16.83% versus 18.87% ± 19.14%, *P* = 0.131) and Proteobacteria (6.95% ± 5.52% versus 16.84% ± 16.89%, *P* = 0.007) showed higher abundances in the RSA group (Fig. 3h and Table S1). Lactobacillus spp. (8.65% ± 16.83% versus 7.80% ± 13.39%, *P* = 0.670) in the UfM also showed a tendency toward lower abundance in the RSA group (Figure S3i and S3k). Four genera, Anaerobacillus (0% ± 0% versus 1.56% ± 5.79%, *P* = 0.011), Erysipelothrix (0% ± 0.01% versus 1.00% ± 2.67%, *P* = 0.011), Bacillus (0.76% ± 0.94 versus 0.25% ± 0.39, *P* = 0.012), and Hydrogenophilus spp. (0.41% ± 0.46% versus 0.48% ± 0.96%, *P* = 0.032), showed significantly different abundances in the UfM, while nonsignificant differences were observed in the EnM between the RSA and control groups (Fig. 3e to h, Tables S1 and S2).

### Functional annotation of the bacterial communities in the RSA and control groups.

**FIG 4:**
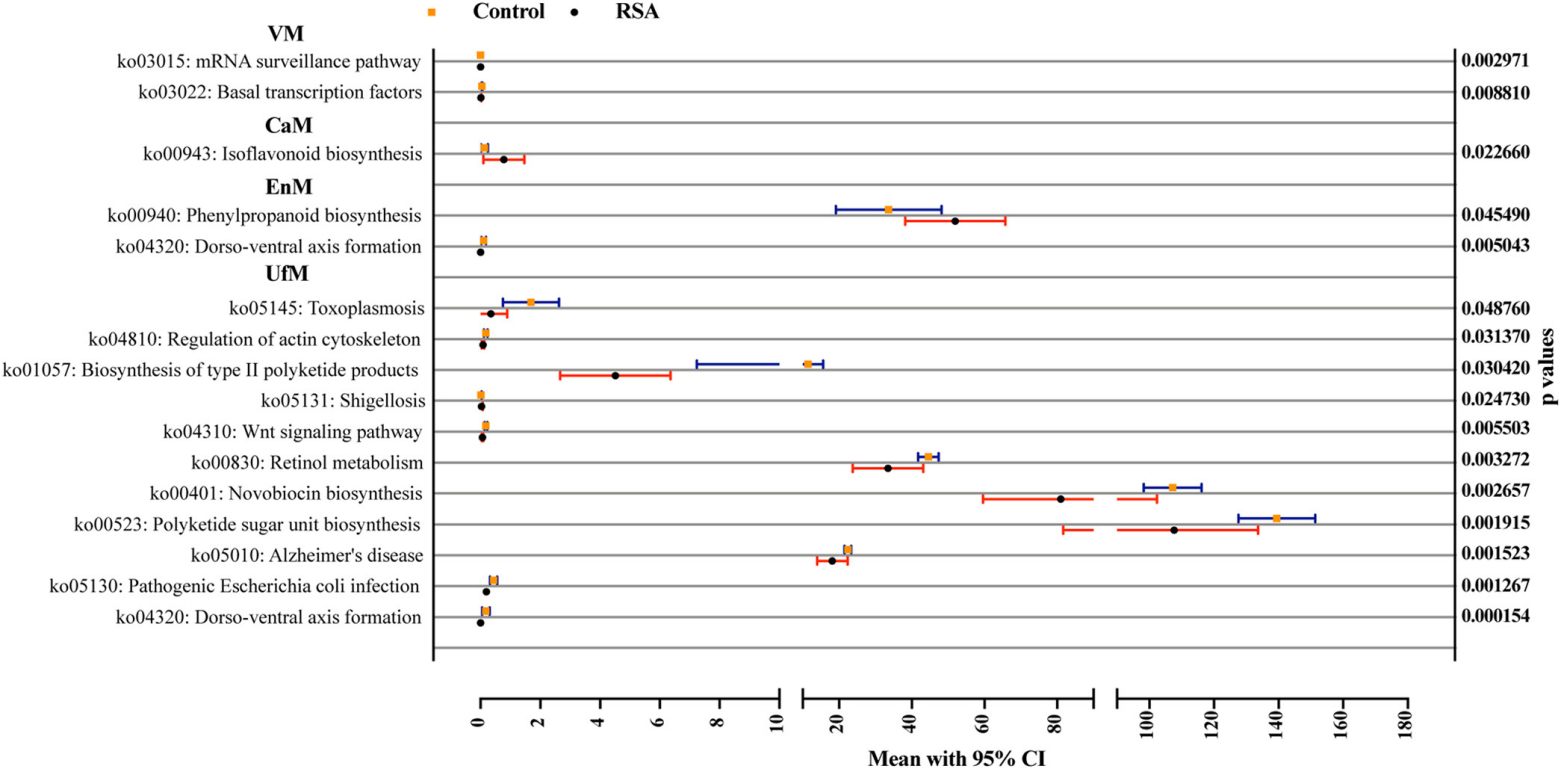
Functional divergence analysis of samples in the KEGG database between the control and RSA groups. *P* values were determined by a two-tailed Mann–Whitney U test, and data are presented as the means with 95% confidence intervals (95% CIs).

As shown in Fig. 4, microbial functions in the vagina, cervix and uterine cavity were predicted by Phylogenetic Investigation of Communities by Reconstruction of Unobserved States (PICRUSt), and the metabolic pathways with significant differences between groups were determined by Kyoto Encyclopedia of Genes and Genomes (KEGG). Functional divergence analysis revealed two pathways in the VM (mRNA surveillance pathway and basal transcription factors), one pathway in the CaM (isoflavonoid biosynthesis), two pathways in the EnM (phenylpropanoid biosynthesis and dorsoventral axis formation), and 11 pathways in the UfM (e.g., dorsoventral axis formation, pathogenic Escherichia coli infection and Wnt signaling pathway) with significant differences.

### Correlations among and alterations in the microbiota from the vagina, cervix and uterine cavity.

The alpha diversity was significantly increased along the lower to upper female reproductive tract, as the richness and diversity increased in both groups (Figure S2a, b, e, and f in the supplemental material). PCoA and beta diversity analysis also showed distinct differences between the microbiota in the lower and upper female reproductive tracts in both groups (Figure S2c, d, g, and h, Table S3). Based on the results in Figure S2i and l, the relative abundances of *Lactobacillus*, *Bifidobacterium*, *Atopobium* and *Gardnerella* spp. showed a sharply decreasing trend, while other bacterial taxa, such as *Bacillus*, *Schlegelella*, *Anaerobacillus*, *Erysipelothrix*, *Halomonas*, *etc.*, showed an opposite trend from the lower genital tract to the upper genital tract in both groups. A total of 215 common OTUs in the control group and 208 common OTUs in the RSA group were also found in different samples along the female genital tract (Figure S2j and m). Network analysis was performed to find the inherent patterns of co-currence or co-exclusion of specific microbial communities driven by spatiotemporal changes and environmental processes. The microbiota in the control group displayed a more complex network than the RSA group but most nodes showed positive correlations along the female reproductive tract in both groups (Figure S2k and n).

### Comparison of the microbiota between the two types of uterine samples.

To investigate the differences in microbial signatures between the two types of uterine samples, further analysis of the uterine microbiota in the RSA and control groups was performed. The Chao1 and Shannon indices of the EnM and UfM showed no statistically significant differences in the control and RSA groups, respectively (Figure S3a and S3d in the supplemental material). Regarding beta diversity, PCoA showed that in the control group, the EnM and UfM samples highly overlapped, and PERMANOVA based on Bray–Curtis dissimilarity showed no distinct difference in the comparison of the EnM and UfM (*P* = 0.281) (Fig. S3b and c). However, a different comparison result was shown in the case of the RSA group. PCoA of the RSA group suggested that the UfM and EnM samples were scattered in a relatively greater fashion (Figure S3e). As shown in Figure S3f, a significant difference between the EnM and UfM in the RSA group was found (*P* = 0.001). The overlapping area shown in the Venn diagram indicates the common OTUs between the corresponding groups. There were 3767 and 3446 common OTUs between the 2 groups of samples in the control and RSA groups, respectively (Fig. S3g and j).

Lactobacillus spp. could be found in the uterine cavity with low relative abundance (<10%). In the control group, the relative abundances of Lactobacillus (4.08% ± 10.06% versus 8.65% ± 16.83%, *P* = 0.017), Acinetobacter (0.24% ± 0.27% versus 0.63% ± 0.73%, *P* = 0.020), and *Vibrio* spp. (0.03% ± 0.05% versus 0.31% ± 0.65%, *P* = 0.032) in the EnM were significantly lower than those in the UfM (Figure S3h and i in the supplemental material). In the RSA group, the relative abundances of Lactobacillus (2.51% ± 4.00% versus 7.80% ± 13.39%, *P* = 0.016), Acinetobacter (3.11% ± 13.68% versus 3.91% ± 15.79%, *P* = 0.044), Cutibacterium (0.91% ± 0.54% versus 2.28% ± 2.68%, *P = *0.043), Bacteroides (0.09% ± 0.15% versus 1.01% ± 3.02%, *P* = 0.023), Halomonas (0.12% ± 0.19% versus 0.75% ± 1.87%, *P* = 0.005), and Muribaculaceae spp. (0.10% ± 0.14% versus 0.71% ± 1.46%, *P* = 0.042) in the EnM were significantly higher than those in the UfM (Figure S3k and l).

As shown in Figure S3m and n in the supplemental material, the relative abundances of microbiota constituents from the kingdom to genus level with significant differences between groups were described by the logarithmic scores of the linear discriminant analysis (LDA). Different uterine cavity samples displayed with different expression of bacterial taxa. At the genus level, 8 types of differentially abundant genera in the UfM (e.g., Nesterenkonia, Lactobacillus, Acinetobacter, Vibrio spp. and so on) and 2 genera (Sphingobacterium and Magnetovibrio spp.) in the EnM with an LDA score >2 were found in the control group (Figure S3m in the supplemental material). Highly abundant bacterial taxa with an LDA score >2 in the RSA group included 13 genera (*RB41*, Weissella, Bacteroides, Cutibacterium, Acinetobacter spp. and so on) in the UfM and 3 genera (Saccharopolyspora, Ruminiclostridium, and Tepidimonas spp.) in the EnM.

### Measurement of human Th1/Th2/Th17 cytokine levels.

**FIG 5:**
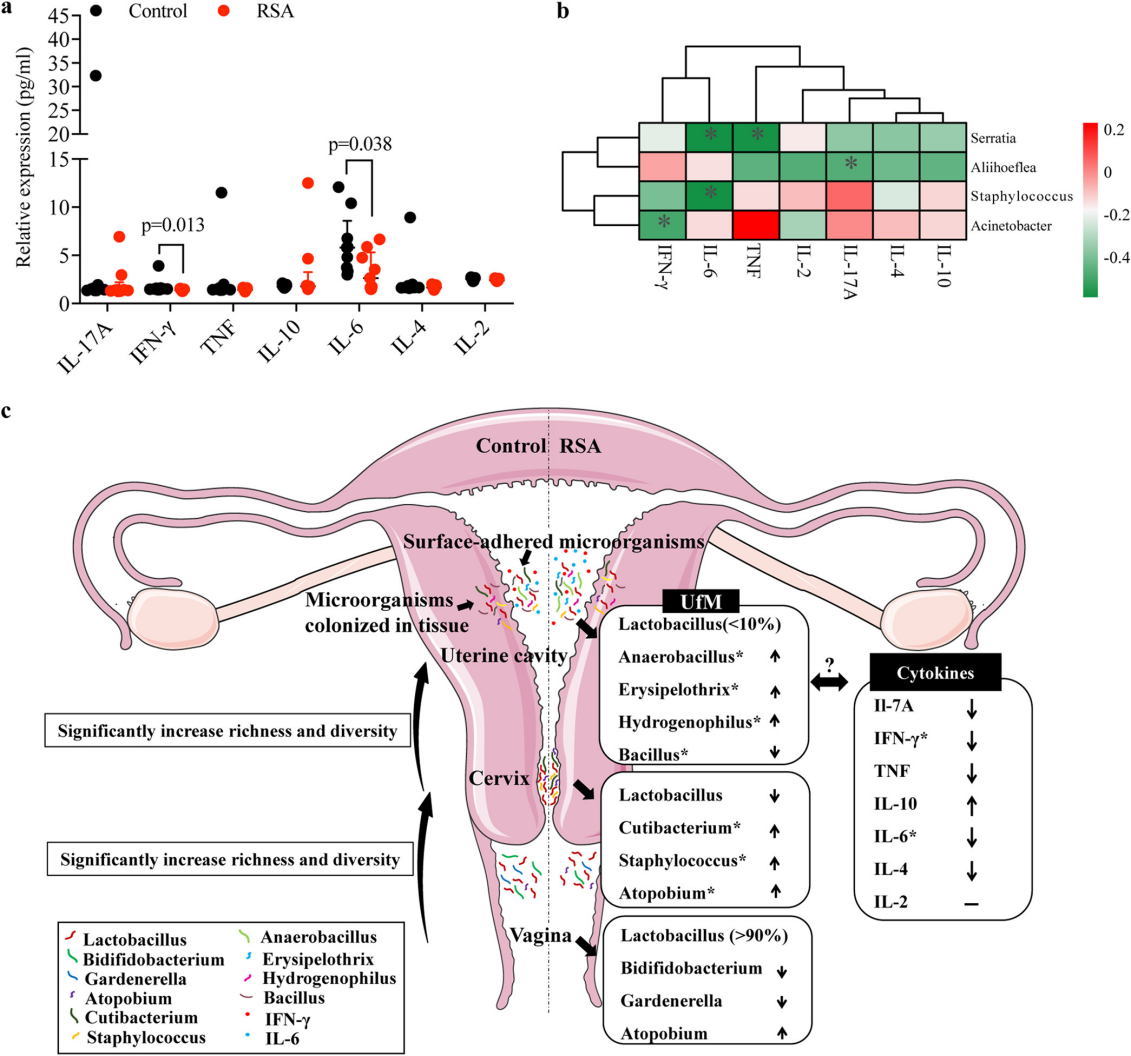
Expression of IL-17A, IFN-γ, TNF, IL-10, IL-6, IL-4 and IL-2 in uterine lavage fluid and its relationship with the microbiota from uterine lavage fluid. Spearman’s correlation analysis among the 15 most abundant bacterial genera in UfM and the cytokine level; only results with significant differences are shown. Data are presented as medians with IQRs (a) and (b). In c, summary results of the alterations of the microbiota within the female reproductive tract in RSA patients.

Nine patients for each group were included to investigate alterations in human inflammatory cytokine levels in uterine lavage fluid. Statistical analysis of the clinical information of the included patients showed no differences between the two groups (Table S4 in the supplemental material). Human Th1/Th2/Th17 cytokines, including IL-17A, IFN-γ, TNF, IL-10, IL-6, IL-4, and IL-2, in uterine lavage fluid were quantified and analyzed. As shown in Fig. 5a, the expression levels of IFN-γ and IL-6 in the RSA group were significantly lower than those in the control group (*P* = 0.013 and *P* = 0.038, respectively). Spearman’s correlation analysis between the top 15 most abundant bacterial taxa in the UfM and human Th1/Th2/Th17 cytokines in uterine lavage fluid were performed. Statistically significant correlations between Aliihoeflea and IL-17A, Acinetobacter and IFN-γ, Serratia and TNF, and Staphylococcus and Serratia and IL-6 were found (Fig. 5b).

## DISCUSSION

This study was the first attempt to systematically characterize microbial dysbiosis in both the lower and upper reproductive tracts in RSA patients. Alterations in microbial biodiversity in the vagina, cervix and uterine lavage fluid were found in RSA patients. The microbiota composition differed markedly from the lower genital tract to the uterine cavity, and the microbiota in the uterine cavity also distinctly varied between endometrial tissue and uterine lavage fluid in the RSA group. Furthermore, alterations in the microbiota in the uterine cavity could be associated with altered cytokine levels, which might be a risk factor for RSA pathogenesis. However, this is only a preliminary study; validation of the findings with a larger sample size of patients and investigation of the underlying mechanism are in need of further research.

RSA, as a clinical challenge with largely unknown factors, has imposed heavy psychiatric and financial burdens on families and society (1). Despite advancements in the understanding of its etiologies, clinical treatment does not always yield substantial improvement due to rising incidence rate and complex etiopathogenesis of RSA (11). A moderate amount of evidence suggests that chronic endometritis is associated with the occurrence of RSA and that chronic endometritis treatment can improve the pregnancy outcomes of RSA (16–18). Since chronic endometritis is commonly caused by infection (19), it is particularly important to subsequently investigate the microbes along the female genital tract, especially the upper female genital tract, in RSA. Research on the microbiota in the female reproductive tract has been conducted for a long time, but many of the relationships with the occurrence of diseases are unknown. In recent years, many studies on the VM in RSA have been successively published (4, 11–13), but relevant studies on the microbiota of the upper female genital tract are scarce.

To further clarify the specific differences in the microflora along the female reproductive tract between the RSA and control groups, the diversity and compositions at the genus level were determined in this study. In contrast to previous results (12, 13), our data showed that the VM in the RSA group did not exhibit significant differences in alpha diversity in this study but showed a significant alteration in beta diversity. For the VM at the genus level, no significant difference between these two groups was detected, but the beneficial microorganisms Bifidobacterium spp. showed a tendency toward decreasing abundance in the RSA group, while the abundance of Atopobium spp. increased in the RSA group. Bifidobacterium strains with a long history as probiotics have been demonstrated to have markedly higher abundance in healthy women (20). Consistent with a previous study (12), Gardnerella spp. in our study also showed higher abundance in the control group. The imbalance of bacterial vaginitis-related microbes, including Gardnerella and Atopobium spp., has been reported in RSA patients and can result in elevated peripheral and uterine natural killer cell counts (10–12). The same trend was also seen in the CaM, which exhibited a significant shift in beta diversity. For the CaM, a trend toward decreasing abundance of Lactobacillus in the RSA group was found. Lactobacillus is the dominant genus in the vagina and cervix in healthy women, and its reduced abundance may cause an infection state and be related to infertility (8). Similar to those in the VM, Atopobium spp. in the cervix also showed a significant increase in abundance in the RSA group, which further demonstrated their possible pathogenicity in the occurrence of RSA. Two other genera, Cutibacterium and Staphylococcus spp., have been previously discovered in the placenta and were considered a potential core microbe in the uterine cavity (21, 22). A significant difference of pH value in the control and RSA groups was not detectable, probably because of the status of asymptomatic microecological disorder in the patients enrolled. This assumption was made because the patients with acute genital tract inflammation, such as bacterial vaginosis and vulvovaginal candidiasis, or human papillomavirus infection were excluded in this study.

Distinct bacterial community structures were observed in the uterine cavity in the RSA group. No dominant bacteria were found in either group, and a multimicrobial system in the uterine cavity was further confirmed in this study. In this study, only the UfM was found to exhibit significant beta diversity, while the EnM did not differ markedly between the control and RSA groups. Upon comparison of the UfM between the RSA and control groups, several bacterial taxa with significantly higher relative abundances in RSA group were found, including Anaerobacillus, Erysipelothrix, and Hydrogenophilus spp. Consistent with previous findings, Anaerobacillus spp. was also found to have a distinctly higher abundance in endometrial tissue that was CD138 positive (23). Since the proportion rate of chronic endometritis in the RSA group was significantly higher than that in the control group, an increased abundance of Anaerobacillus spp. was entirely plausible in this study. Erysipelothrix spp. have also been isolated from the uterus and placenta in animals, but they are mostly considered a primary or opportunistic pathogen (24). Hydrogenophilus spp., as thermophilic aerobic betaproteobacteria, are commonly found in geothermal environments (25). Consistent with a previous study (9), our study also indicated that pH results of uterine lavage fluid between the groups were not significantly different.

To our knowledge, this was the first report to find Hydrogenophilus spp. in the uterine cavity, and their function in the uterine cavity remains unknown. In addition, a significant reduction in the abundance of *Bacillus* spp. in uterine lavage fluid was also found in the RSA group. Bacillus spp. have been reported in the uterine microbiota of healthy postpartum dairy cows, and the presence of Bacillus pumilus could result in higher mRNA expression of interleukin 1α, IL-6, IL-8, and CXCL1-3, causing an inflammatory reaction in the endometrium (26, 27). Consistent with a previous study (14), a significant difference in beta diversity between the UfM and EnM was found in the RSA group. Interestingly, this phenomenon was not found in the control group. In the control group, the profile of the UfM was similar to that of the EnM. Although common OTUs were shared in these samples, significant differences still existed in microbial community structure in different uterine samples. There is an assumption that some microorganisms are likely to be on the luminal surface, while other bacteria colonize deeper endometrial tissue. Together with the above evidence, these results suggested that significant alterations in the surface-adhered microorganisms might be the potential cause of RSA. This evidence also indicated that different methods for collecting the microbiota should be considered to provide a more thorough and wider comprehensive exploration of the microbiota in the uterine cavity.

Alterations in the uterine microbiota compared with that of controls usually result in altered cytokine levels (15). Dramatic drops in IFN-γ and IL-6 levels were observed in the RSA group in this study, and Spearman’s correlation analysis demonstrated that the abundances of several bacterial taxa were weakly but statistically significantly negatively related to the expression levels of Th1/Th2/Th17 cytokines (Fig. 5c). However, the bacterial taxa associated with the altered cytokines showed no significant differences in the RSA group and vice versa, probably because of the small sample size used in the cytokine analysis. However, the interactions of the microbiota and cytokines should be noted, and further studies with larger sample sizes are warranted to explore this relationship.

Metabolic divergence analysis revealed that the dorsoventral axis formation pathway in the uterus was remarkably different between the RSA and control groups. A case report indicated that a spontaneously aborted male fetus with fatal defects probably arose from an abnormal dorsoventral axis formation process (28). Dorsoventral axis formation is an important process for determining the development of embryo limbs and trunks and is regulated by Wnt signaling (29, 30). Alterations in the Wnt signaling pathway were found in the RSA group, and it is well known that the Wnt signaling pathway is essential for the implantation and decidualization processes (31). Hence, these functional alterations differed considerably in the control and RSA groups, and the difference might exert an important effect on the occurrence of RSA. Pathogenic Escherichia coli infection was also distinctly different between the control and RSA groups. It has been reported that Escherichia coli infection of the female reproductive tract is an important factor contributing to severe uterine inflammation and disturbance of the profile of cytokines, including leukotrienes, TNF, and IL-6 (32, 33). Overall, shifts in the microbial community structure might result in changes in metabolic and inflammatory status.

In this study, we also reported that the microbiota composition was significantly altered from the vagina to the uterine cavity, with increased richness and increased diversity in both groups. Highly abundant Lactobacillus, Atopobium, and Gardnerella spp. were found in the VM and CaM in the RSA group, while the abundances of Acinetobacter, Anaerobacillus, Erysipelothrix, Bacillus, and Hydrogenophilus spp. were significantly elevated in the uterine microbiota in the RSA group. These results indicated that the key microbiota involved in pathogenic processes were different in the vagina, cervix and uterine cavity. Hence, suitable treatment regimens in the clinic for microbiota disorders in the female reproductive tract should be provided, with regulation at different locations. However, further rigorous clinical trials with lager sample size are still needed to clarify specific clinical treatment strategies.

## CONCLUSION

In conclusion, the evidence in this study suggested that significant alterations in the microbial profile of the vagina, cervix and uterine cavity were present in RSA patients, and the key microbiota in different locations involved in pathogenic processes were different, which indicated that different treatments should be considered. The microbiota in the uterine cavity was distinctly different from that in the lower genital tract, and significant differences between the two types of uterine microbiota were also found in both groups. In general, our study demonstrated that the uterine cavity was colonized with some microbial community structures and that their disorder might result in several differentially abundant pathways and fluctuations in IFN-γ and IL-6 levels, which might represent potential pathogenic pathways causing RSA. Hence, the pathogenesis of the microbiota along the female reproductive tract in RSA merits further investigation to provide a more theoretical and experimental basis for clinic applications.

## MATERIALS AND METHODS

### Study design.

To compare the differences in the microbiota between the RSA group (*n* = 25) and the control group (*n* = 25), different samples, including vaginal secretions, cervical secretions, intrauterine lavage fluid, and endometrial tissue, were collected from patients on their receptive days (5–7 days after luteinizing hormone surge, LH + 5–7). To more comprehensively understand the characteristics of intrauterine microorganisms, endometrial tissue samples and uterine lavage fluid were collected simultaneously. The ovulation date was determined by transvaginal ultrasound and/or urine LH test paper and further confirmed by serum progesterone quantification on the day of sampling. Chronic endometritis was diagnosed according to the pathological results with immunohistochemical staining of CD138 positive plasma cells (34).

### Patient enrollment and recruitment.

This study was approved by the Ethics Committee of Reproductive Medicine, the Third Hospital of Peking University (No. 2019SZ-067). All patients in this study were enrolled according to the inclusion and exclusion criteria.

Inclusion criteria:

1. Patients whose informed consent was obtained after having been duly informed of the nature of the study and who voluntarily agreed to participate after being fully aware of the potential risks, benefits and any discomfort involved.
2. Maternal age: 18–42 years.
3. BMI: 18.5–30.0 kg/m^2^ (both inclusive).
4. Nonpregnant, with a regular menstrual cycle.
5. Negative serological tests for hepatitis B surface antigen (HBsAg), hepatitis C virus (HCV), and human immunodeficiency virus (HIV).
6. No antibiotic use, vaginal drug use or cervical treatment in the past 30 days and no sexual activity in the past 2 weeks.
7. Two or more consecutive spontaneous abortions occurred in the individuals in the RSA group, and infertile patients due to male factors were included in the control group.

Exclusion criteria:

1. Patients with chromosome abnormalities according to the analysis of the chromosome karyotypes in peripheral blood.
2. Any pathological finding affecting the endometrial cavity preliminarily detected by transvaginal ultrasound, such as adenomyosis, submucosal myomas or intramural myomas > 4 cm, or hydrosalpinx, must have been previously confirmed and operated on at least 3 months before the endometrial samples were obtained (note: patients were allowed to participate if the pathology was corrected before performing any study procedure).
3. Polycystic ovary syndrome and hypothyroidism, hyperthyroidism, diabetes, antithrombotic syndrome or Sjogren's syndrome.
4. Patients with acute genital tract inflammation, such as bacterial vaginosis and vulvovaginal candidiasis, or HPV infection.
5. Illness or unstable medical conditions that may have put the patient at risk of safety and compliance in the study.

### Sample collection and total DNA extraction.

Before sampling, patients were placed in the lithotomy position, and the cervix and vagina were fully exposed with a sterile speculum. The operator wore sterile gloves for sampling and performed the whole sampling process in a sterile situation, and the sampling process is shown in Fig. 1.

Vaginal secretions from the upper one-third of the vaginal sidewalls were obtained with a sterile cotton swab, and the lower rod of the swab was separated into a tube containing sterile transfer medium (4U003S. CN, Copan).

After wiping the secretion from the external opening of the cervix with sterile dry cotton, a flocked cotton swab was placed into the cervix canal, and the vaginal wall was carefully not touched.

According to the standard operating procedure of hysteroscopy examination, the vagina and the external opening of the cervix were disinfected by iodophor after vaginal and cervical canal secretions were collected, and a sterile dry cotton ball was used to clean the disinfectant the surface of the cervix to prevent the disinfectant flowing into the uterine cavity. The inner sheath of an artificial insemination tube (AIC18, Smiths Medical) was then gently introduced trans-cervically into the uterine cavity under the protection of the outer sheath and connected with a 5 mL sterile syringe. Then, 2 mL of sterile normal saline was injected, quickly withdrawn and transferred to an empty sterile tube.

Under the protection of the outer sheath, the endometrial negative pressure catheter was placed into the uterine cavity for suction and curettage. The catheter was removed, and then the endometrial tissue was transferred into a sterile tube.

In this study, negative controls including normal saline and transfer medium were used to exclude potential bacterial contaminations from DNA processing and library preparation. All samples were sealed, put into liquid nitrogen immediately and stored at −80°C before DNA extraction. Then, total bacterial genomic DNA samples were extracted using an OMEGA Soil DNA Kit (M5635-02, Omega Bio-Tek) according to the manufacturer’s instructions and stored at −20°C prior to further analysis. Samples were labeled by abbreviations as follows: VM is the abbreviation for the vaginal microbiota, CaM is the abbreviation for the cervical microbiota, UfM is the abbreviation for the microbiota of uterine lavage fluid and EnM is the abbreviation for the microbiota of endometrial tissue. C is the abbreviation for the control group, and A is the abbreviation for the RSA group.

### 16S rDNA amplicon pyrosequencing.

PCR amplification of the bacterial 16S rRNA gene V3–V4 region was performed using the forward primer 338F (5′-ACTCCTACGGGAGGCAGCA-3′) and the reverse primer 806R (5′-GGACTACHVGGGTWTCTAAT-3′). Sample-specific 7-bp barcodes were incorporated into the primers for multiplex sequencing. The PCR components contained 5 μL of buffer (5×), 0.25 μL of Fast PFU DNA polymerase (5 U/μL), 2 μL (2.5 mM) of dNTPs, 1 μL (10 μM) of each forward and reverse primer, 1 μL of DNA template, and 14.75 μL of ddH_2_O. Thermal cycling consisted of initial denaturation at 98°C for 5 min, followed by 25 cycles consisting of denaturation at 98°C for 30 s, annealing at 53°C for 30 s, and extension at 72°C for 45 s, with a final extension of 5 min at 72°C. PCR amplicons were purified with Vazyme VAHTSTM DNA Clean Beads (N411-03, Vazyme) and quantified using a Quant-iT PicoGreen dsDNA assay kit (P7589, Invitrogen). After the individual quantification step, amplicons were pooled in equal amounts, and paired-end 2 × 250 bp sequencing was performed using the Illumina NovaSeq platform with a NovaSeq 6000 SP reagent kit (500 cycles) at Shanghai Personal Biotechnology Co., Ltd. (Shanghai, China).

### Sequence analysis.

Microbiome bioinformatic analysis was performed with QIIME2 2019.4, with slight modifications, according to the official tutorials (https://docs.qiime2.org/2019.4/tutorials/) (35). Briefly, raw sequence data were demultiplexed using the demux plugin followed by primer trimming with the cutadapt plugin. Sequences were then quality filtered, denoised, and merged, and chimeras were removed using the DADA2 plugin (36). Nonsingleton amplicon sequence variants (ASVs) were aligned with mafft and used to construct a phylogeny with fasttree2 (37). Alpha diversity metrics (Chao1 and Shannon) and beta diversity metrics (Bray–Curtis dissimilarity) were estimated using the diversity plugin, with samples rarefied to 60,000 sequences per sample. Taxonomy was assigned to ASVs using the classify-sklearn naive Bayes taxonomy classifier in the feature-classifier plugin against the SILVA release 132 database (38, 39).

### Bioinformatic analysis.

Sequence data analysis was mainly performed using the QIIME2 and R packages (v3.2.0). OTU-level alpha diversity indices, such as the Chao1 richness estimator and Shannon diversity index, were calculated using the OTU table in QIIME2. Beta diversity analysis was performed to investigate the structural variation in microbial communities across samples using Bray–Curtis metrics (40, 41) and visualized via PCoA and unweighted pair-group method with arithmetic means (UPGMA) hierarchical clustering (42). The significance of the differentiation of microbiota structure among groups was assessed by PERMANOVA using QIIME2. The taxonomy compositions and abundances were visualized using MEGAN and GraPhlAn (43, 44). A Venn diagram was generated to visualize the shared and unique OTUs among samples or groups using the R package “VennDiagram” based on the occurrence of OTUs across samples/groups regardless of their relative abundances (45). Taxon abundances at the phylum, class, order, family, genus and species levels were statistically compared among samples or groups by Metastats (46). LDA effect size was performed to detect differentially abundant taxa across groups using the default parameters (47). The generalization error was estimated using 10-fold 2 cross-validation. The expected “baseline” error was also included, which was obtained by a classifier that simply predicts the most common category label. Microbial functions were predicted by PICRUSt2 in the KEGG (https://www.kegg.jp/) database (48).

### Cytokine testing.

Uterine lavage fluid (RSA: *n* = 9; Control: *n* = 9) was collected, and inflammatory cytokine levels were measured via a BD CBA Human Th1/Th2/Th17 Cytokine Kit (560484, BDPMG). According to the manufacturer’s instructions, cytokine capture beads, including those for IL-17A, IFN-γ, TNF, IL-10, IL-6, IL-4, and IL-2, were mixed with 50 μL samples and then incubated with PE-conjugated detection antibodies at room temperature for 3 h. The mixture was then analyzed on a BD FACSCelesta (Becton, Dickinson), and data were analyzed using FlowJo software.

### Statistical analysis.

GraphPad Prism version 7.0 (GraphPad Software) and SPSS version 26.0 were used for statistical analysis. One-way analysis of variance followed by Tukey’s *post hoc* test was used to evaluate the statistical significance of differences for multiple comparisons, and Student's *t* test was used for comparison of two-group data. For comparing categorical data, a χ^2^ test was performed, with Fisher’s correction when necessary. For nonparametric tests, the two-tailed Mann–Whitney U test was used to evaluate statistical significance between two groups, and the Kruskal–Wallis test followed by Dunn’s *post hoc* analysis was used for three or more groups. Data are shown as the means ± SEMs/SDs or as the medians with interquartile ranges. Spearman’s correlation analysis between two factors was performed. *P* < 0.05 was considered statistically significant.

### Data availability.

Sequence reads for the 194 specimens included in this study have been deposited in the National Center for Biotechnology Information (NCBI) under reference number PRJNA774109 and PRJNA813906.
