# Supplementary material for: An Altered Microbiota in the Lower and Upper Female Reproductive Tract of Women with Recurrent Spontaneous Abortion
Source: Microbiol Spectr. 2022 May 23;10(3):e00462-22. doi: 10.1128/spectrum.00462-22 (PMC9241707; doi:10.1128/spectrum.00462-22)
Supplement: SUPPLEMENTAL FILE 1 — Fig. S1 to S4. Download spectrum.00462-22-s001.pdf, PDF file, 5.7 MB [file spectrum.00462-22-s001.pdf]

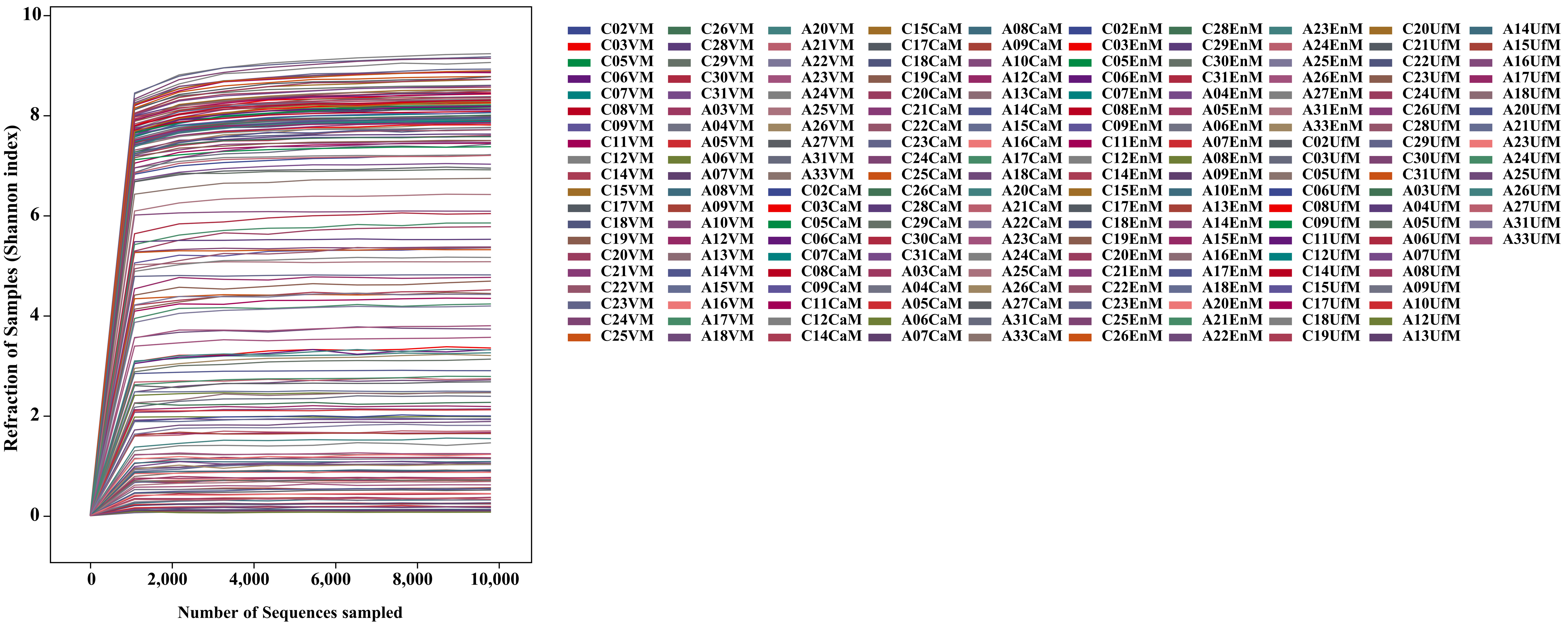

Supplementary Figure 1. Refraction curves of the sequenced samples.

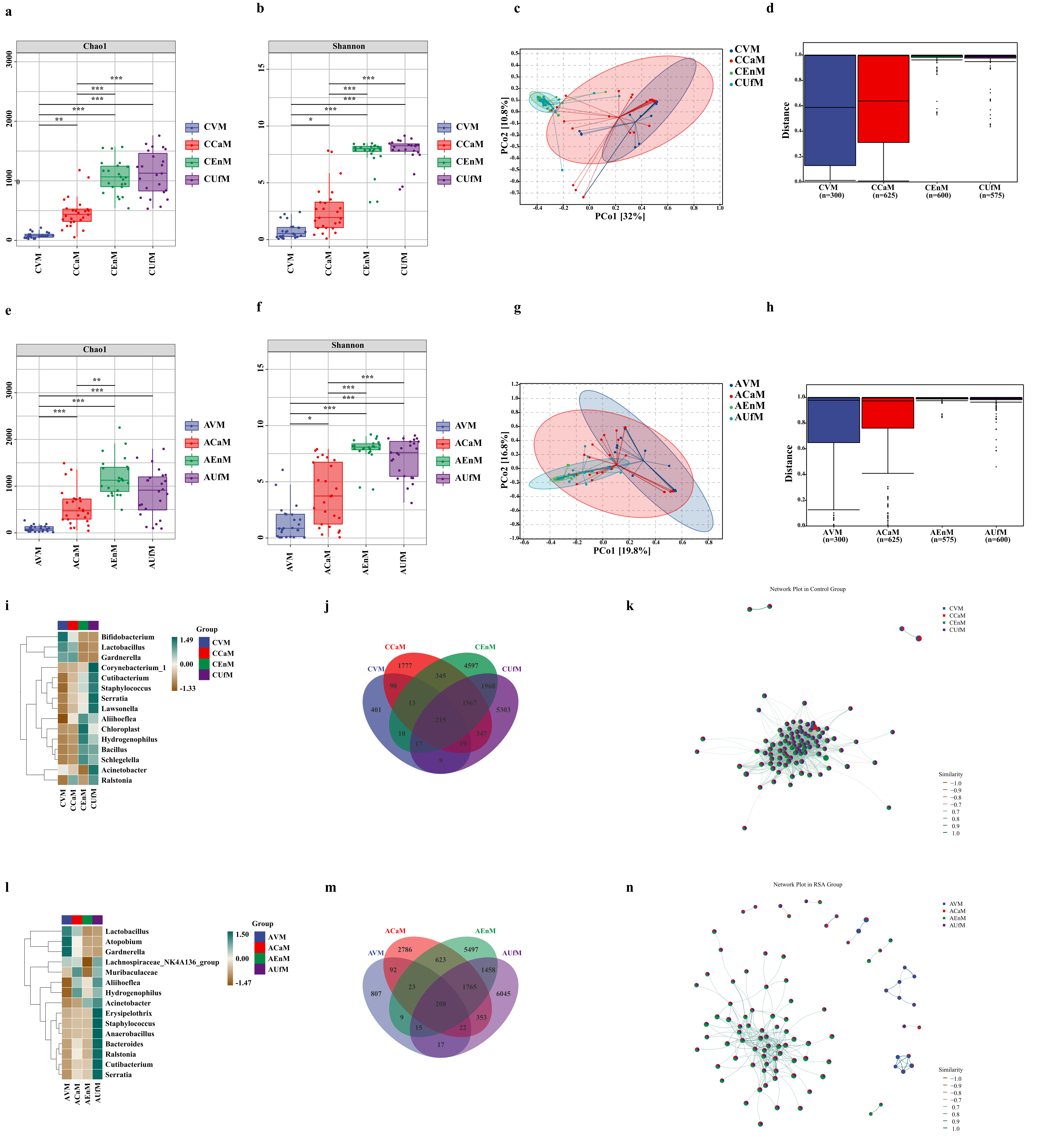

Supplementary Figure 2. Correlations among and alterations in the microbiota from the vagina, cervix and uterine cavity.Alpha diversity including Chao 1 and Shannon index of all samples from the control (a-b) and RSA groups (e-f). PCoA plots and beta diversity based on Bray–Curtis metrics of all samples from the control (c-d) and RSA groups (g-h). Heatmaps of all samples at the genus level in the control (i) and RSA groups (l). The samples were clustered via UPGMA according to the European distance of the species composition data, and the results were presented as heatmaps. Each block represents the average abundance of genera in each sample, and the deeper the color is, the higher the genus abundance. Venn diagram of all samples in the control (j) and RSA groups (m). Each color block represents a group; the overlapping area represents the common OTUs. Network diagram of microbial ecology from the control (k) and RSA groups (n). Each node (or vertex) represents an OTU or taxa in the community; The node size is positively proportional to its abundance (in log2 (CPM / N)). The connecting line between two points represents the distribution trend of positive or negative correlation between two connected points. P values were determined by Kruskal–Wallis test followed by Dunn's post-hoc test, and data are presented as medians with IQRs. *p <0.05, ** p <0.01, ***p <0.001.

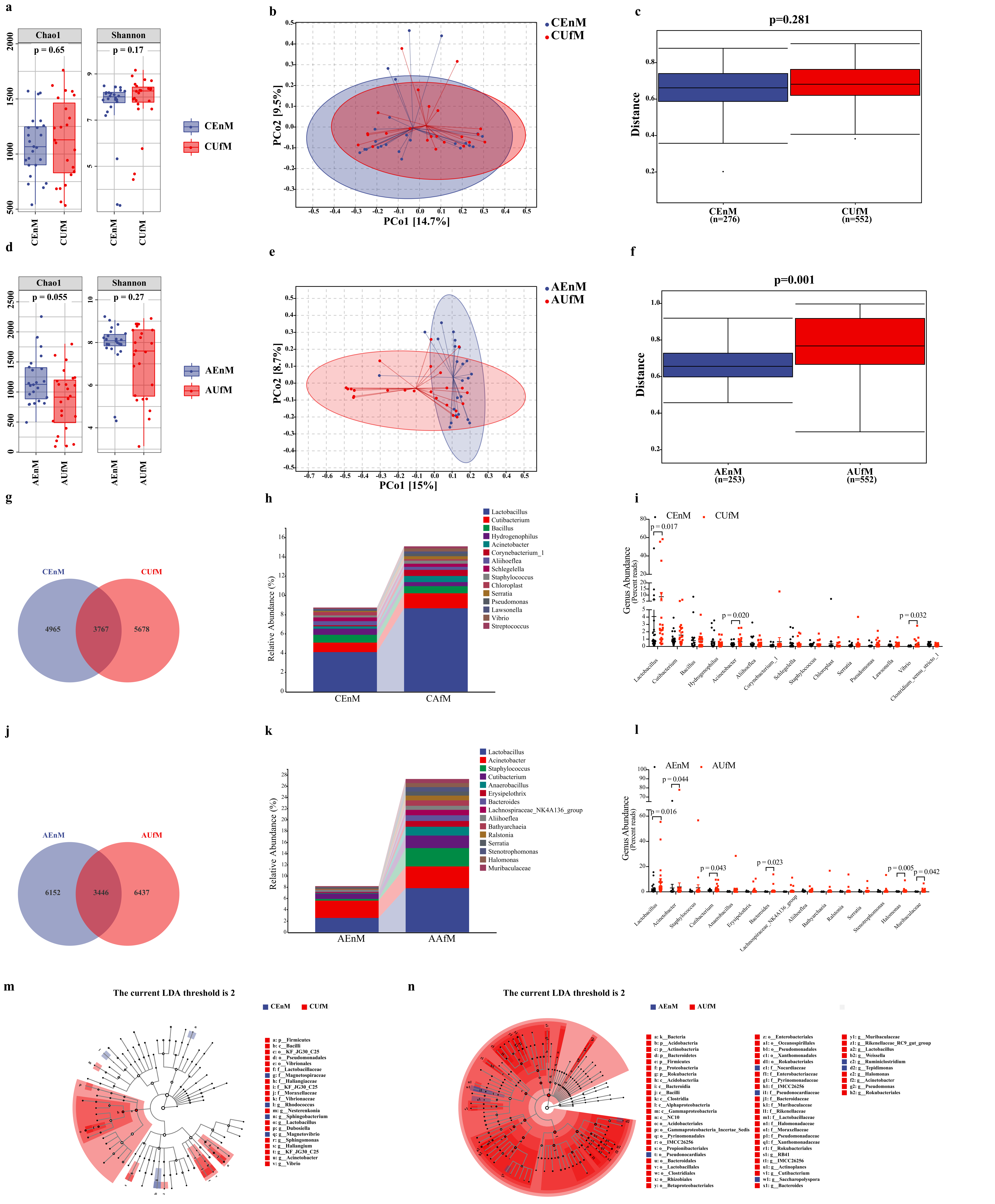

Supplementary Figure 3. Comparison of the microbiota between the two types of uterine samples. Alpha diversity including Chao 1 and Shannon index of uterine microbiota via 2 sampling methods from the control (a) and RSA groups (d). PCoA plots and beta diversity based on Bray–Curtis metrics of uterine microbiota from 2 sampling methods of the control (b-c) and RSA groups (e-f). Venn diagram of OTUs for uterine microbiota via 2 sampling methods from the control (g) and RSA groups (j). Taxonomic classification and the top 15 bacterial genus abundances were compared among 2 communities of uterine microbiota via different sampling methods from the control (h-i) and RSA groups (k-l). P values were determined by a two-tailed Mann–Whitney U test, and data are presented as the means with s.e.m. LEfSe analysis of the control (m) and RSA groups (n) for different group samples and bacterial taxa with an LDA score >2 is shown. k: kingdom, p: phylum, c: class, o: order, f: family, g: genus. P values were determined by a two-tailed Mann–Whitney U test, and data are presented as medians with IQRs.

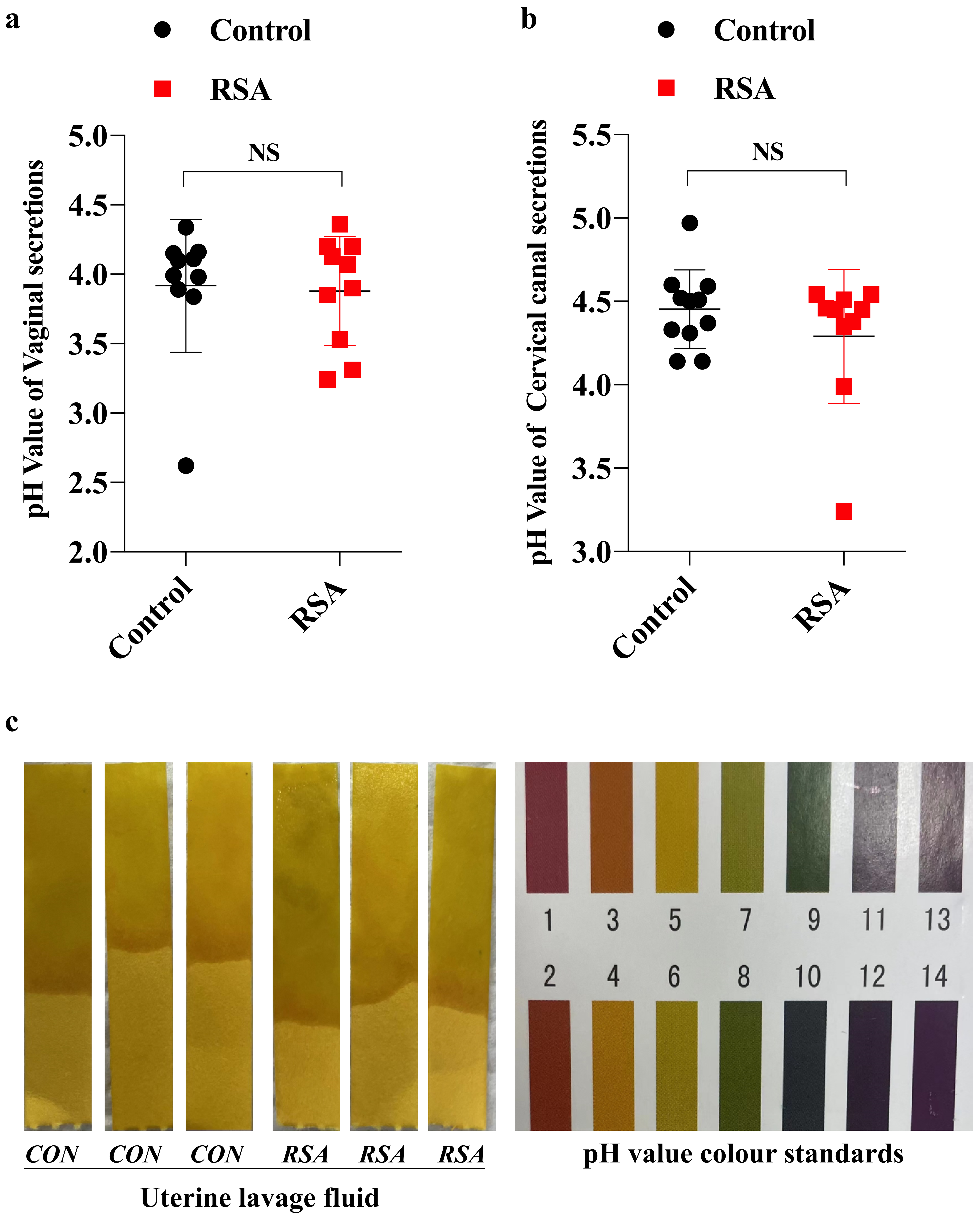

Supplementary Figure 4. pH tests of vaginal secretions (a), cervical canal secretions (b), and uterine lavage fluid (c).
